# Vertebral hemangiosarcoma as a cause of spinal cord compression in a dog

**DOI:** 10.29374/2527-2179.bjvm009425

**Published:** 2026-02-10

**Authors:** Fernanda Barthelson Carvalho de Moura, Pedro Pol Ximenes, Silmara Santos Lazzaretti, Emerson Gonçalves Martins de Siqueira, John Kastelic, Carlos Eduardo Fonseca Alves, Didier Quevedo Cagnini

**Affiliations:** 1 Veterinary Clinic Department, School of Veterinary Medicine and Animal Science, São Paulo State University, Botucatu, SP, Brazil; 2 School of Veterinary Medicine and Animal Science, São Paulo State University, Botucatu, SP, Brazil; 3 Veterinary Surgery and Reproduction Department, School of Veterinary Medicine and Animal Science, São Paulo State University (UNESP), Botucatu, SP, Brazil; 4 Faculty of Veterinary Medicine, University of Calgary, Calgary, Canada

**Keywords:** spinal cord compression, immunohistochemistry, primary neoplasm, compressão espinhal, imunohistoquímica, neoplasia primária

## Abstract

Primary central nervous system (CNS) neoplasia is relatively rare in most domestic animal species, except dogs and cats, being directly associated with aging. On the other hand, hemangiosarcoma, lymphoma, mammary carcinoma and melanoma are the most common secondary tumors affecting CNS. They usually represent metastasis, and less frequently arising from nearby areas that infiltrate or compress CNS normal tissue, resulting in neurological clinical signs. This case report documents a 10-year-old female spayed Pinscher presenting with severe thoracolumbar pain and paraplegia due to a primary vertebral hemangiosarcoma (HSA). CT revealed a lytic lesion in the L1 vertebra causing dorsal displacement and spinal cord compression. Surgical removal of the neoplasm involved a bloc vertebral resection and stabilization with screws, cement, and soft tissue reconstruction, leading to marked clinical improvement. Histopathology confirmed an infiltrative primary bone HSA with positive immunohistochemical staining for endothelial marker. Postoperative adjuvant therapy with metronomic cyclophosphamide and propranolol was associated with extended survival and maintained quality of life over 13 months. This report underscores the importance of combined surgical and chemotherapeutic management for primary vertebral HSA in dogs, and this neoplasm as a less likely differential diagnosis for local invasive compressive neoplasms in CNS in dogs.

## Resumo

A neoplasia primária do sistema nervoso central (SNC) é relativamente rara na maioria das espécies de animais domésticos, exceto cães e gatos, estando diretamente associada ao envelhecimento. Por outro lado, o hemangiossarcoma, o linfoma, o carcinoma mamário e o melanoma são os tumores secundários mais comuns que afetam o SNC. Eles geralmente representam metástases e, menos frequentemente, surgem de áreas próximas que infiltram ou comprimem o tecido normal do SNC, resultando em sinais clínicos neurológicos. Este relato de caso documenta uma fêmea de 10 anos de idade, castrada, da raça Pinscher, apresentando dor toracolombar intensa e paraplegia devido a um hemangiossarcoma (HSA) vertebral primário. A TC revelou uma lesão lítica na vértebra L1, causando deslocamento dorsal e compressão da medula espinhal. A remoção cirúrgica da neoplasia envolveu a ressecção em bloco da vértebra e a estabilização com parafusos, cimento e reconstrução de tecidos moles, levando a melhora clínica acentuada. A histopatologia confirmou um HSA ósseo primário infiltrativo com imuno-histoquímica positiva para o marcador endotelial. A terapia adjuvante pós-operatória com ciclofosfamida metronômica e propranolol foi associada ao aumento da sobrevida e à manutenção da qualidade de vida por 13 meses. Este relato ressalta a importância do tratamento cirúrgico e quimioterápico combinado para a HSA vertebral primário em cães, e essa neoplasia como um diagnóstico diferencial menos provável para neoplasias compressivas invasivas locais no SNC em cães.

## Introduction

Canine hemangiosarcoma (HSA), a malignant tumor originating from endothelial cells, frequently metastasizes and does not respond well to chemotherapy (Mulia et al., 2021; Parchman & Crameri, 1989). These tumors have a propensity for primary development in the spleen, liver, heart, skin, and muscle (Pérez-Martínez et al., 2016; Reis et al., 2017; Stock et al., 2011) with site-dependent clinical signs (Pérez-Martínez et al., 2016).

However, primary vertebral HSA is rare (Mulia et al., 2021; Pérez-Martínez et al., 2016); only 2 primary HSA case reports have been published, but no therapeutic approach was explored. This study reports the imaging, surgical, macroscopic, microscopic, and clinical findings of a vertebral HSA in a Brazilian pinscher dog.

## Case description

A 10-year-old female spayed pinscher dog weighing 4 kg was referred to the veterinary teaching hospital. The dog presented with a lytic punctual lesion in the first lumbar vertebra. There was a history of severe back pain and paraplegia, with normal spinal reflexes and absence of deep pain in the hind limbs. The owner noted that the dog otherwise had no previous health issues.

A complete blood count (CBC) revealed a normocytic and normochromic anemia (red blood cell count: 3.70 × 10^6^ µL, reference range (RR): 5.5–8.5 × 10^6^ µL; hemoglobin concentration: 8.70 g/dL, reference range (RR): 12.0–18.0 g/dL; hematocrit: 26%, RR: 37–55%; mean corpuscular volume: 77.3 fL, RR: 60–77 fL; mean cellular hemoglobin concentration: 33.4%, RR: 32–36%); leukocytosis (24 600/μL, RR: 5500 to 19 500/μL) with neutrophilia (22 881/μL; RR: 1925 to 14 625/μL). Serum biochemical evaluation revealed increased activities of alanine aminotransferase (109 IU/L, RR: 21–73 IU/L) and alkaline phosphatase (611 IU/L, RR: 20–156 IU/L).

**Figure 1:**
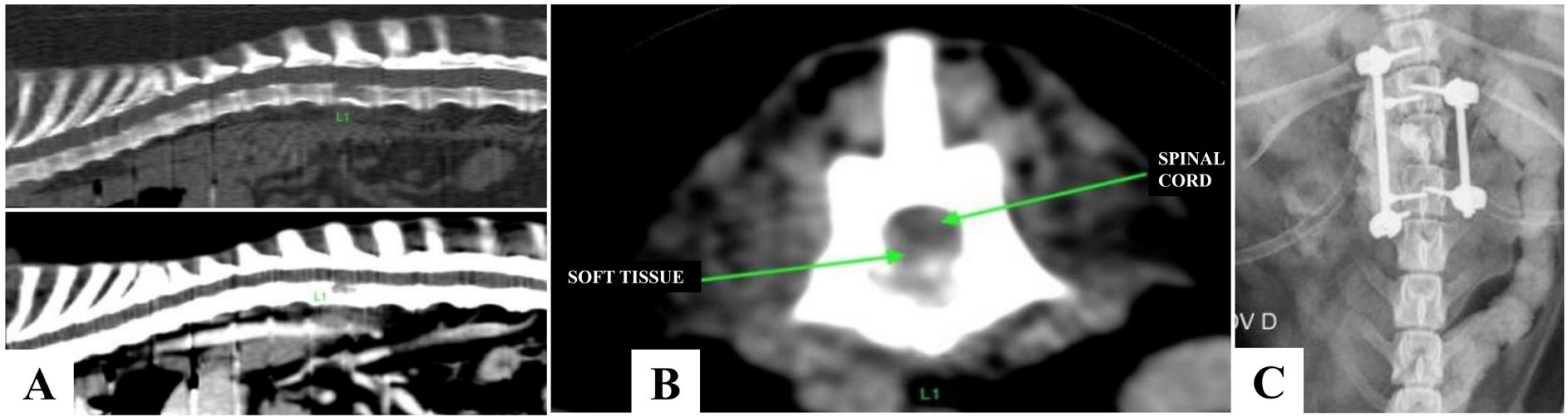
Photograph of a 10-year-old female spayed Pinscher dog with vertebral column hemangiosarcoma. A - Lateral view of the neoplasm in L1 vertebra. B - Coronal view of the same neoplasm invading the spinal canal via soft tissue in the right ventral and ventrolateral positions to the spinal cord, causing dorsal displacement and spinal cord compression. C - Vertebral body reconstruction with 2 blocked screws on the left and right sides of the vertebrae.

Computed tomography (CT) of the thoracolumbar spinal cord and lumbosacral segments T5 to S2 was performed before and after IV administration of contrast medium. Four study series were made, including the scan series and 3 cross-sectional series (thickness, 2 mm). Multiple reconstructions of the series were done. The CT findings were compatible with a neoplasm in L1 vertebra; invasion of the spinal channel by soft tissue content was noted in the right ventral and ventrolateral positions, causing dorsal displacement and spinal cord compression (Figure 1). The possibility of a differential diagnosis of osteomyelitis was also considered.

The dog underwent surgery to remove the tumor. The surgery allowed soft tissue removal in the vertebral channel through placement of a left polyaxial screw to prevent space collapse after vertebrectomy. Finally, vertebral body reconstruction was performed with 2 blocked screws on the right side, and bone cement with polymethylmethacrylate (PMMA) and polyaxial screws placed on the right side of the vertebra. Muscle closure was performed with poliglecaprone 2.0 (Figure 1). The dog was transferred to an urgent and intensive care unit for post-surgery monitoring. Analgesia included methadone 0.3 mg/kg q12h IV, dipyrone 25 mg/kg q8h IV, and dexamethasone 0.1 mg/kg q24h SC. At 2 wk after surgery, metronomic chemotherapy was initiated with cyclophosphamide 15 mg/mm^2^ q24h PO and propranolol 2 mg/kg q24h PO for 120 d.^1,2^ The patient was monitored every 3 mo.

After surgery, the dog developed chronic diarrhea. A coproparsitology exam was negative for parasites. However, stool consistency improved following a diet change to Royal Canin Gastrointestinal Dog Food. Based on abdominal ultrasonography, the dog’s left renal lymph node was 3.58 x 2.19 cm before surgery, but it was 7.2 x 4.2 cm at 2 mo after surgery. At the time of submission of this case report (13 mo after surgery), the dog is alive and receiving cyclophosphamide 15 mg/mm^2^ q24h PO and propranolol 2 mg/kg q24h PO, with no substantial side effects and good quality of life.

**Figure 2:**
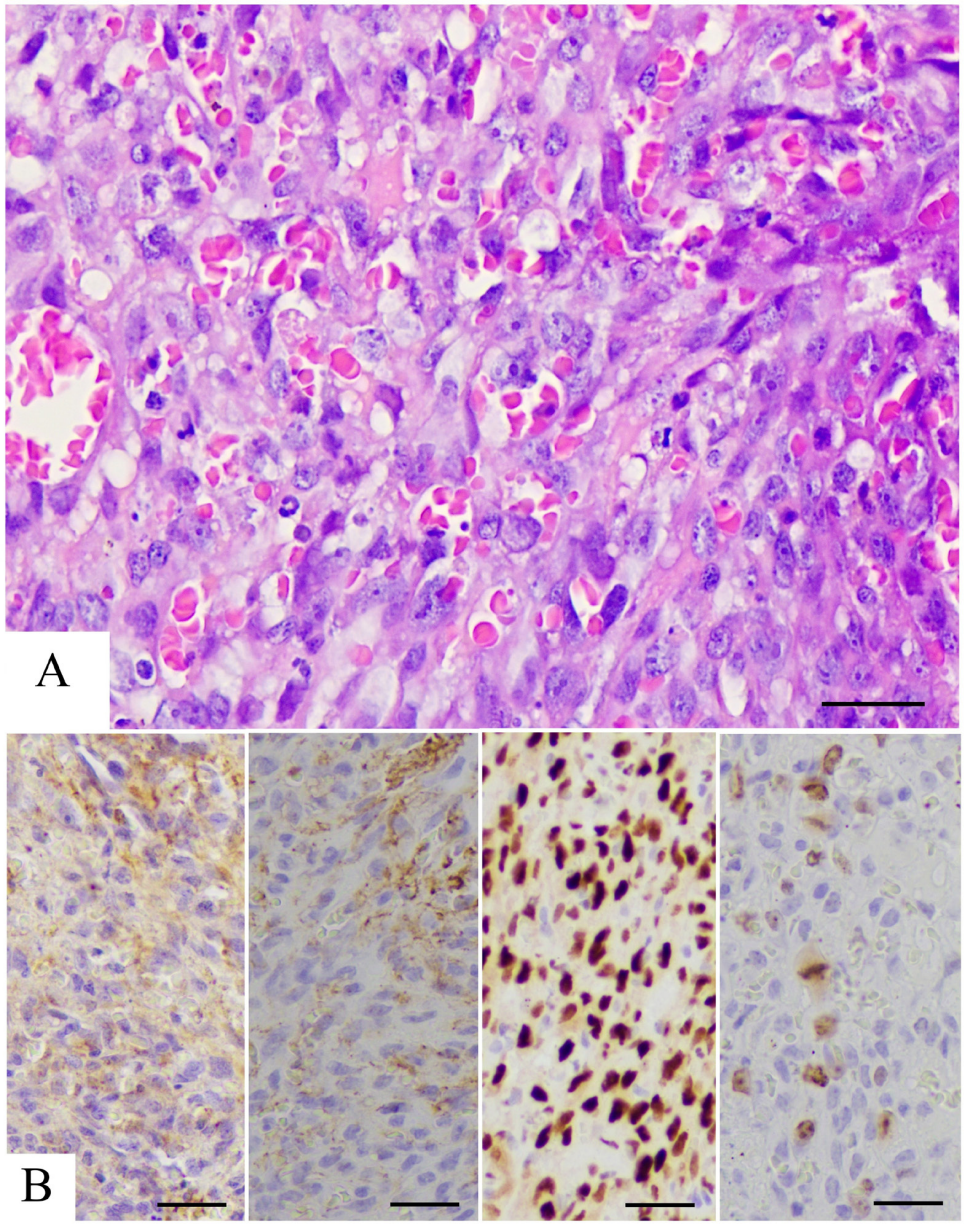
Histopathology of a vertebral hemangiosarcoma in a 10-year-old female spayed Pinscher dog. (A) Infiltrative, nondelimited neoplastic proliferation of mesenchymal cells inside the bone lacunae, forming vessels filled with red blood cells. H&E stain, bar=50 µm**.** (B) Immunohistochemical panel showing positive neoplastic cells for VIII-Factor, CD31, ERG, and Ki67 (left to right). Bar=50 µm.

Excised tissue had blackish to whitish fragments and was 0.2 × 1.6 cm. Histopathology revealed portions of well-differentiated bone surrounded by bone marrow cells. Moreover, there was normal skeletal muscle and fat tissue, and multifocal hemorrhagic areas. Furthermore, mesenchymal cells' infiltrative, nondelimited neoplastic proliferation was observed inside bone lacunae, forming vessels filled with red blood cells. The cells had poorly delimited eosinophilic cytoplasm, rounded to elongate nuclei with loose chromatin, and single to multiple nucleoli, with accentuated nuclear pleomorphism and anisokaryosis. Three mitotic figures were observed in 2 fields at 400x-magnification (2.37 mm^2^) (Figure 2). Immunohistochemistry revealed that neoplastic cells were positive for factors VIII, CD31, ERG, and Ki67 (20%) (Figure 2). Therefore, the definitive diagnosis was hemangiosarcoma.

## Discussion

This report describes a vertebral HSA case in a dog presenting deep back pain and paraplegia in a thoracolumbar syndrome, due to vertebral compression caused by a mass. Diagnostic imaging, surgery, histopathology, and immunohistochemistry contributed to the final diagnosis (De Nardi et al., 2023).

Primary central nervous system (CNS) neoplasia is relatively rare in most domestic animal species, except dogs and cats, being directly associated with aging. Among them, meningiomas are the most common, representing 50% of all primary CNS tumors in dogs, and up to 85% in cats. Conversely, HSA, lymphoma, mammary carcinoma, and melanoma are the most common secondary tumors affecting CNS^4^. They usually represent metastasis from a distant primary neoplasia, and less frequently arising from nearby areas that infiltrate or compress CNS normal tissue, resulting in neurological clinical signs (De Nardi et al., 2023).

Few new therapies for HSA have been advanced in recent years, and surgery still represents the best therapeutic option, with chemotherapy providing an adjuvant effect. Surgery plus low-dose continuous chemotherapy improved survival to ~ 6 mo on average (Hammer et al., 1991; Lana et al., 2007). In the present study, surgery enabled removal of the vertebral compression (and accompanying pain) caused by the neoplasm, greatly improving the dog’s quality of life. Enlargement of the left renal lymph node may have been caused by gastrointestinal chronic inflammation or HSA metastasis, although this is not often a metastasis site for this neoplasm (De Nardi et al., 2023). Metronomic chemotherapy with cyclophosphamide and propranolol increases survival and neoplasm-free time; this therapy was chosen based on good responses in dogs with advanced HSA with metastasis (Elmslie et al., 2008; Terauchi et al., 2023).

Endothelial neoplasms like HSA are rare in humans. The most common types are nasopharyngeal angiofibroma, hemangioma, and angiosarcoma (Nelson & Thompson, 2007). Human angiosarcoma occurs in the skin and mammary gland but is most often a secondary lesion in women with breast cancer after radiation treatment (Wang et al., 2020). Existing studies of human angiosarcoma are mostly isolated case reports, offering only low-quality clinical evidence. However, as angiosarcoma in humans is regarded as the counterpart of canine HSA, reporting cases in dogs may motivate further studies of HAS that may be used as a model for human angiosarcoma (Parchman & Crameri, 1989; Ray-Coquard et al., 2015). In addition, further studies on the diagnosis, treatment, and prognosis of vertebral HSA in dogs should provide evidence to better manage this condition and improve quality of life for affected dogs.

## Conclusion

Although cases of hemangiosarcoma in the CNS of dogs are usually metastatic, this neoplasm should be considered as a less likely differential diagnosis for local invasive compressive neoplasms in CNS in the dogs.
